# (*O*,*O*′-Diethyl dithio­phosphato-κ^2^ *S*,*S*′)(hydridotripyrazol-1-ylborato-κ^3^ *N* ^2^,*N* ^2′^,*N* ^2′′^)(triphenyl­phosphine-κ*P*)ruthenium(II)

**DOI:** 10.1107/S160053680803170X

**Published:** 2008-10-09

**Authors:** Hung-Chun Tong, Chih-Yung Chen Hsu, Yao-Ren Liang, Yih Hsing Lo, Chia-Her Lin

**Affiliations:** a Department of Chemical Engineering, Tatung University, Taipei 104, Taiwan; b Department of Chemistry, Chung-Yuan Christian University, Chung-Li 320, Taiwan

## Abstract

Reaction of [Ru(Tp)Cl(PPh_3_)_2_] {where Tp is hydridotri­pyrazol­yl­borate, BH[C_3_H_3_N_2_)_3_)]} with NH_4_[S_2_P(OEt)_2_] in methanol afforded the title compound, [Ru(C_9_H_10_BN_6_)(C_4_H_10_O_2_PS_2_)(C_18_H_15_P)], in which the Ru^II^ ion is in a slightly disorted octa­hedral coordination environment. The [S_2_P(OEt)_2_]^−^ ligand coordinates in a chelating mode with two similar Ru—S bond lengths and a slightly acute S—Ru—S angle. The atoms of both –OCH_2_CH_3_ groups of the diethyl dithio­phosphate ligand are disordered over two sites with approximate occupancies of 0.76 and 0.24.

## Related literature

For related structures, see: Alock *et al.* (1992 ▶); Burrows (2001 ▶); Hidai *et al.* (2000 ▶); Gemel *et al.* (1996 ▶); Jain & Jakkal (1996 ▶); Meno *et al.* (1995 ▶); Pavlik *et al.* (2005 ▶); Sellmann *et al.* (1999 ▶); Slugovc *et al.* (1998 ▶); Vit & Zdrazil (1989 ▶).
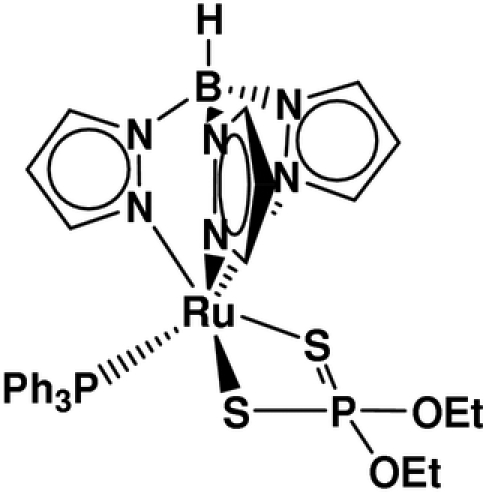


## Experimental

### 

#### Crystal data

[Ru(C_9_H_10_BN_6_)(C_4_H_10_O_2_PS_2_)(C_18_H_15_P)]
*M* *_r_* = 761.59
Monoclinic, 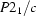

*a* = 12.4408 (2) Å
*b* = 13.7386 (2) Å
*c* = 20.3775 (3) Å
β = 99.676 (1)°
*V* = 3433.36 (9) Å^3^
*Z* = 4
Mo *K*α radiation
μ = 0.71 mm^−1^
*T* = 200 (2) K
0.42 × 0.3 × 0.15 mm

#### Data collection

Nonius KappaCCD diffractometer
Absorption correction: multi-scan (*SORTAV*; Blessing, 1995 ▶) *T*_min_ = 0.755, *T*_max_ = 0.901
24009 measured reflections
6265 independent reflections
5601 reflections with *I* > 2σ(*I*)
*R*_int_ = 0.051

#### Refinement

*R*[*F* ^2^ > 2σ(*F* ^2^)] = 0.036
*wR*(*F* ^2^) = 0.093
*S* = 1.05
6265 reflections
449 parameters
42 restraints
H-atom parameters constrained
Δρ_max_ = 1.43 e Å^−3^
Δρ_min_ = −0.62 e Å^−3^

### 

Data collection: *COLLECT* (Nonius, 1999 ▶); cell refinement: *DENZO* and *SCALEPACK* (Otwinowski & Minor, 1997 ▶); data reduction: *DENZO* and *SCALEPACK*; program(s) used to solve structure: *SHELXS97* (Sheldrick, 2008 ▶); program(s) used to refine structure: *SHELXL97* (Sheldrick, 2008 ▶); molecular graphics: *ORTEP-3 for Windows* (Farrugia, 1997 ▶); software used to prepare material for publication: *WinGX* (Farrugia, 1999 ▶).

## Supplementary Material

Crystal structure: contains datablocks I, global. DOI: 10.1107/S160053680803170X/lh2697sup1.cif

Structure factors: contains datablocks I. DOI: 10.1107/S160053680803170X/lh2697Isup2.hkl

Additional supplementary materials:  crystallographic information; 3D view; checkCIF report

## Figures and Tables

**Table:** 

Ru1—N3	2.086 (2)
Ru1—N1	2.088 (2)
Ru1—N5	2.144 (3)
Ru1—P1	2.3171 (8)
Ru1—S1	2.4540 (8)
Ru1—S2	2.4635 (8)

**Table:** 

N3—Ru1—N1	90.09 (9)
N3—Ru1—N5	83.57 (10)
N1—Ru1—N5	84.88 (10)
N3—Ru1—P1	90.07 (7)
N1—Ru1—P1	90.63 (7)
N5—Ru1—P1	172.20 (7)
N3—Ru1—S1	170.14 (7)
N1—Ru1—S1	93.07 (7)
N5—Ru1—S1	87.41 (7)
P1—Ru1—S1	99.23 (3)
N3—Ru1—S2	94.61 (7)
N1—Ru1—S2	169.78 (7)
N5—Ru1—S2	86.63 (7)
P1—Ru1—S2	98.42 (3)
S1—Ru1—S2	80.86 (3)

**Fig. 1.**
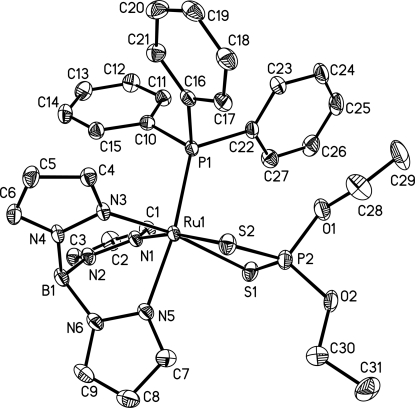
Molecular structure of (I) showing displacement ellipsoids at the 35% level and H atoms having arbitrary radius. The disorder is not shown.

## supplementary crystallographic information

### Comment

The chemistry of transition metal sulfur compounds has attracted interest for their importance in the field of catalysts, metalloenzymes, and materialprecursor (Hidai *et al.*,2000). In recent years there has been an increased interest in ruthenium sulfurcomplexes, in part because of the high catalytic activity of RuS_2_ in various hydrotreating processes (Vit & Zdrazil, 1989). As a part of this development, many examples of ruthenium thiolate complexes have been reported, however, the ruthenium complexes with dithio ligands are relatively rare (Sellmann *et al.*, 1999). On the other hand, ruthenium(II)hydridotripyrazolylborate complexes, Ru(Tp), are of interest for stoichiometricand catalytic transformations of organic molecules (Pavlik *et al.*, 2005). The complex [Ru(Tp)Cl(PPh_3_)_2_] (Alock *et al.*, 1992) has been used as the starting material for the synthesis of several complexes because of its substitutionally labile chloride and phosphines (Burrows, 2001). In order to aquire a better understanding of the coordination chemistry of RuS_2_,we have studied the ruthenium phosphine complex containing the ligands hydrotris(pyrazolyl)borate (Tp) and [NH_4_][S_2_P(OEt)_2_]. Interaction of [Ru(Tp)Cl(PPh_3_)_2_] with [NH_4_][S_2_P(OEt)_2_] in MeOH afforded the title compound {Ru(Tp)(PPh_3_)[S_2_P(OEt)_2_]} (I). The ^31^P NMRspectrum of (I) in CDCl_3_ shows two intense singletsat 50.8 and 105.7 p.p.m., assignable to PPh_3_ and [S_2_P(OEt)_2_],respectively. The FAB mass spectrum of (I) shows the molecular ions {Ru(Tp)(PPh_3_)[S_2_P(OEt)_2_]} with the characteristic isotopic distribution patterns. The crystal structure of (I) was established by X-ray crystallography. In the title compound, the environment about the Ru^II^ ion is slightly distorted octahedral and the bite angle of the Tp ligand produces an average N—Ru—N angle of ca. 86° only slightly distorted from 90°. The three Ru—N(Tp) bond lengths are slightly longer than the average distance of 2.038 Å in other ruthenium Tp complexes (Gemel *et al.*1996; Slugovc *et al.*1998). The [S_2_P(OEt)_2_]^– ^ligand chelates the ruthenium centre with two nearly equal Ru—S bonds and the S—Ru—S angle is slightly acute. The Ru—S bond lengths in (I) are comparable to those in [(*η*^6^-*p*-cymene)Ru{S_2_P(OMe)_2_}(PPh_3_)][BPh_4_][av. 2.4311 (12) Å] with a chelated dithiophosphate ligand (Jain & Jakkal, 1996), but slightly longer than for *cis*-[Ru(S_2_CNEt_2_)_2_(PPh_3_)_2_][av. 2.3952 (5) Å] with chelated dithiocarbamate (Meno *et al.*., 1995). The Ru—P bond length in (I) agrees well with those in related ruthenium(II) complexes with PPh_3_ ligands (Jain & Jakkal, 1996, Meno *et al.*, 1995).

### Experimental

The synthesis of the title compound (I) was carried out as follows. To a solution of [Ru(Tp)Cl(PPh_3_)_2_](3.95 g,4.50 mmol) in MeOH (20 ml), an excess of [NH_4_][S_2_P(OEt)_2_] (1.82 g, 9.00 mmol) were added. The reaction mixture was stirred for a further 8 h at room temperature. The solvent was dried under vacuum and 20 ml of CH_2_Cl_2_ was added to the residue. The product was dissolved in CH_2_Cl_2_ and other salts such as [NH_4_][S_2_P(OEt)_2_] and NH_4_Cl precipitated. After filtration, the solvent was dried under vacuum to give the title compound (I) (3.27 g, 95% yield). Spectroscopic analysis: IR (KBr, cm^-1^): ν(BH)2468 cm^-1.1^H NMR (CDCl_3_, 303 K, d,p.p.m.): d 7.92 (d, *J*_H—H _= 2.3 Hz, 1H, Tp),7.83 (d, *J*_H—H _= 2.3 Hz, 1H, Tp), 7.71 (d, *J*_H—H _=2.3 Hz, 1H, Tp), 7.4–6.9 (m, Tp, Ph), 6.83 (d, *J*_H—H _= 2.3 Hz,1*H*, Tp), 5.81 (d, *J*_H—H _= 2.2 Hz, 1H, Tp), 5.66 (d, *J*_H—H_= 2.2 Hz, 1H, Tp), 5.63 (t, *J*_H—H _= 2.2 Hz, 1H, Tp), 5.54(t, *J*_H—H _= 2.2 Hz, 1H, Tp), 4.16 (q, *J*_H—H _=7.2 Hz, 2H, OCH_2_), 3.11 (q, *J*_H—H _= 7.2 Hz, 2H, OCH_2_),1.32(t, *J*_H—H _= 7.2 Hz, 3H, CH_3_), 0.79 (t, *J*_H—H_= 7.2 Hz, 3H, CH_3_).^13^C NMR (CDCl_3_,303 K, d, p.p.m.): 146.7–104.6 (m, PPh_3_, Tp), 60.6,61.4 (d, OCH_2_, ^2^*J*_P—C_ = 10 Hz), 15.5,15.9 (d, OCH_2_*C*H_3_, ^3^*J*_P—C_= 8.4 Hz). ^31^P NMR (CDCl_3_, 303 K, d,p.p.m.): d 105.7 (PS_2_), 50.9 (s, PPh_3_). MS (*m*/*z*,Ru102): 762.2 (*M*^+^), 500.1(*M*^+^ - PPh_3_). Anal. Calcdfor C_31_H_35_BN_6_O_2_P_2_RuS_2_:*C*, 48.89; H, 4.63; N, 11.03. Found: C, 48.73; H,4.61; N, 11.02. The bright-yellow crystalsof (I) for X-ray structure analysis were obtained by recrystallization of the crude product from dichloromethane-hexane.

### Refinement

H atoms were placed in idealized positions and constrained to ride on their parent atoms, with C—H = 0.93 - 0.97 Å and *U*_iso_(H) = 1.2 or 1.5*U*_eq_(C), B—H = 0.98 Å and *U*_iso_(H) = 1.2*U*_eq_(C). The atoms of both -OCH_2_CH_3_ groups of the diethyldithiophosphato ligand are disordered over two sites with refined occupancies of 0.764 (3) and 0.236 (3).

### Crystal data

**Table:**

[Ru(C_9_H_10_BN_6_)(C_4_H_10_O_2_PS_2_)(C_18_H_15_P)]	*F*(000) = 1560
*M**_r_* = 761.59	*D*_x_ = 1.473 Mg m^−^^3^
Monoclinic, *P*2_1_/*c*	Mo *K*α radiation, λ = 0.71073 Å
Hall symbol: -P 2ybc	Cell parameters from 32408 reflections
*a* = 12.4408 (2) Å	θ = 2.0–25.4°
*b* = 13.7386 (2) Å	µ = 0.71 mm^−^^1^
*c* = 20.3775 (3) Å	*T* = 200 K
β = 99.676 (1)°	Prism, yellow
*V* = 3433.36 (9) Å^3^	0.42 × 0.3 × 0.15 mm
*Z* = 4	

### Data collection

**Table:**

Nonius KappaCCD diffractometer	6265 independent reflections
Radiation source: fine-focus sealed tube	5601 reflections with *I* > 2σ(*I*)
graphite	*R*_int_ = 0.051
Detector resolution: 9 pixels mm^-1^	θ_max_ = 25.4°, θ_min_ = 2.4°
CCD rotation images, thick slices scans	*h* = −11→14
Absorption correction: multi-scan (SORTAV; Blessing, 1995)	*k* = −16→16
*T*_min_ = 0.755, *T*_max_ = 0.901	*l* = −24→24
24009 measured reflections	

### Refinement

**Table:**

Refinement on *F*^2^	Primary atom site location: structure-invariant direct methods
Least-squares matrix: full	Secondary atom site location: difference Fourier map
*R*[*F*^2^ > 2σ(*F*^2^)] = 0.036	Hydrogen site location: inferred from neighbouring sites
*wR*(*F*^2^) = 0.093	H-atom parameters constrained
*S* = 1.05	*w* = 1/[σ^2^(*F*_o_^2^) + (0.0373*P*)^2^ + 5.0939*P*] where *P* = (*F*_o_^2^ + 2*F*_c_^2^)/3
6265 reflections	(Δ/σ)_max_ = 0.001
449 parameters	Δρ_max_ = 1.43 e Å^−^^3^
42 restraints	Δρ_min_ = −0.62 e Å^−^^3^

### Special details

**Table:**

Geometry. All e.s.d.'s (except the e.s.d. in the dihedral angle between two l.s. planes) are estimated using the full covariance matrix. The cell e.s.d.'s are taken into account individually in the estimation of e.s.d.'s in distances, angles and torsion angles; correlations between e.s.d.'s in cell parameters are only used when they are defined by crystal symmetry. An approximate (isotropic) treatment of cell e.s.d.'s is used for estimating e.s.d.'s involving l.s. planes.
Refinement. Refinement of *F*^2^ against ALL reflections. The weighted *R*-factor *wR* and goodness of fit *S* are based on *F*^2^, conventional *R*-factors *R* are based on *F*, with *F* set to zero for negative *F*^2^. The threshold expression of *F*^2^ &gt; σ(*F*^2^) is used only for calculating *R*-factors(gt) *etc*. and is not relevant to the choice of reflections for refinement. *R*-factors based on *F*^2^ are statistically about twice as large as those based on *F*, and *R*- factors based on ALL data will be even larger.

### Fractional atomic coordinates and isotropic or equivalent isotropic displacement parameters (Å^2^)

**Table:**

	*x*	*y*	*z*	*U*_iso_*/*U*_eq_	Occ. (<1)
Ru1	0.685497 (18)	0.068530 (16)	0.255762 (11)	0.02384 (9)	
S1	0.79384 (7)	0.17427 (6)	0.19498 (4)	0.0388 (2)	
S2	0.74769 (7)	−0.05385 (6)	0.18210 (4)	0.03638 (19)	
P1	0.81369 (6)	0.03972 (6)	0.35016 (4)	0.02612 (17)	
P2	0.84695 (7)	0.05178 (7)	0.16235 (4)	0.0369 (2)	
N1	0.6227 (2)	0.18566 (18)	0.30252 (12)	0.0289 (5)	
N2	0.5136 (2)	0.18862 (18)	0.30454 (13)	0.0324 (6)	
N3	0.57667 (19)	−0.02517 (18)	0.29151 (12)	0.0272 (5)	
N4	0.47495 (19)	0.00904 (18)	0.29637 (13)	0.0301 (6)	
N5	0.5506 (2)	0.09368 (19)	0.17715 (13)	0.0314 (6)	
N6	0.4510 (2)	0.11145 (19)	0.19433 (14)	0.0339 (6)	
C1	0.6649 (3)	0.2667 (2)	0.33239 (16)	0.0351 (7)	
H1A	0.7383	0.2835	0.3384	0.042*	
C2	0.5841 (3)	0.3225 (3)	0.35311 (19)	0.0454 (9)	
H2A	0.5922	0.3822	0.3749	0.054*	
C3	0.4901 (3)	0.2709 (2)	0.33460 (17)	0.0406 (8)	
H3A	0.4212	0.2897	0.3417	0.049*	
C4	0.5802 (3)	−0.1169 (2)	0.31240 (15)	0.0307 (7)	
H4A	0.6401	−0.1579	0.3140	0.037*	
C5	0.4826 (3)	−0.1432 (2)	0.33140 (17)	0.0396 (8)	
H5A	0.4647	−0.2030	0.3480	0.048*	
C6	0.4181 (3)	−0.0622 (2)	0.32047 (17)	0.0371 (7)	
H6A	0.3469	−0.0571	0.3284	0.045*	
C7	0.5363 (3)	0.0975 (3)	0.11105 (17)	0.0410 (8)	
H7A	0.5909	0.0872	0.0858	0.049*	
C8	0.4285 (3)	0.1189 (3)	0.08504 (19)	0.0493 (9)	
H8A	0.3978	0.1263	0.0405	0.059*	
C9	0.3772 (3)	0.1267 (3)	0.13905 (18)	0.0445 (9)	
H9A	0.3037	0.1403	0.1378	0.053*	
C10	0.7727 (2)	0.0899 (2)	0.42642 (15)	0.0295 (6)	
C11	0.8439 (3)	0.1437 (2)	0.47297 (15)	0.0351 (7)	
H11A	0.9154	0.1535	0.4667	0.042*	
C12	0.8085 (3)	0.1827 (3)	0.52858 (17)	0.0419 (8)	
H12A	0.8566	0.2186	0.5593	0.050*	
C13	0.7026 (3)	0.1686 (3)	0.53855 (17)	0.0437 (8)	
H13A	0.6789	0.1958	0.5754	0.052*	
C14	0.6322 (3)	0.1137 (3)	0.49359 (17)	0.0427 (8)	
H14A	0.5614	0.1026	0.5009	0.051*	
C15	0.6662 (3)	0.0751 (2)	0.43786 (16)	0.0354 (7)	
H15A	0.6177	0.0389	0.4077	0.042*	
C16	0.8413 (2)	−0.0886 (2)	0.37420 (16)	0.0300 (6)	
C17	0.8813 (3)	−0.1501 (2)	0.32978 (17)	0.0389 (8)	
H17A	0.8871	−0.1272	0.2876	0.047*	
C18	0.9128 (3)	−0.2447 (3)	0.34695 (19)	0.0441 (8)	
H18A	0.9395	−0.2845	0.3165	0.053*	
C19	0.9043 (3)	−0.2796 (2)	0.40899 (19)	0.0446 (9)	
H19A	0.9265	−0.3427	0.4210	0.054*	
C20	0.8628 (3)	−0.2207 (3)	0.45330 (19)	0.0464 (9)	
H20A	0.8553	−0.2448	0.4949	0.056*	
C21	0.8320 (3)	−0.1256 (2)	0.43639 (17)	0.0387 (8)	
H21A	0.8049	−0.0863	0.4670	0.046*	
C22	0.9557 (2)	0.0851 (2)	0.35834 (15)	0.0314 (7)	
C23	1.0452 (3)	0.0291 (3)	0.38653 (17)	0.0386 (8)	
H23A	1.0344	−0.0335	0.4015	0.046*	
C24	1.1505 (3)	0.0660 (3)	0.39243 (19)	0.0470 (9)	
H24A	1.2096	0.0273	0.4102	0.056*	
C25	1.1676 (3)	0.1587 (3)	0.37228 (19)	0.0515 (10)	
H25A	1.2381	0.1833	0.3767	0.062*	
C26	1.0798 (3)	0.2161 (3)	0.34532 (19)	0.0494 (9)	
H26A	1.0912	0.2794	0.3318	0.059*	
C27	0.9746 (3)	0.1791 (3)	0.33844 (17)	0.0406 (8)	
H27A	0.9160	0.2180	0.3202	0.049*	
O1	0.9685 (2)	0.0388 (3)	0.20330 (16)	0.0485 (8)	0.764 (3)
C28	1.0361 (4)	−0.0397 (4)	0.1920 (3)	0.0657 (16)	0.764 (3)
H28A	1.0254	−0.0524	0.1446	0.079*	0.764 (3)
H28B	1.0124	−0.0971	0.2134	0.079*	0.764 (3)
C29	1.1497 (4)	−0.0264 (6)	0.2152 (5)	0.079 (2)	0.764 (3)
H29A	1.1884	−0.0839	0.2059	0.118*	0.764 (3)
H29B	1.1619	−0.0149	0.2623	0.118*	0.764 (3)
H29C	1.1753	0.0283	0.1929	0.118*	0.764 (3)
O1A	0.9713 (4)	0.0320 (9)	0.1572 (5)	0.0479 (13)	0.236 (3)
C29A	1.1270 (19)	−0.061 (2)	0.2317 (18)	0.079 (2)	0.236 (3)
H29D	1.1592	−0.0565	0.2779	0.118*	0.236 (3)
H29E	1.1827	−0.0527	0.2047	0.118*	0.236 (3)
H29F	1.0928	−0.1229	0.2229	0.118*	0.236 (3)
C28A	1.0470 (15)	0.0148 (15)	0.2162 (8)	0.0648 (19)	0.236 (3)
H28C	1.0029	0.0108	0.2511	0.078*	0.236 (3)
H28D	1.0878	0.0750	0.2241	0.078*	0.236 (3)
O2	0.8764 (3)	0.0499 (3)	0.08958 (13)	0.0458 (9)	0.764 (3)
C30	0.7920 (4)	0.0556 (4)	0.0330 (2)	0.0476 (13)	0.764 (3)
H30A	0.7472	0.1124	0.0366	0.057*	0.764 (3)
H30B	0.7459	−0.0017	0.0311	0.057*	0.764 (3)
C31	0.8393 (18)	0.0618 (10)	−0.0271 (6)	0.068 (3)	0.764 (3)
H31A	0.7821	0.0649	−0.0650	0.102*	0.764 (3)
H31B	0.8835	0.0054	−0.0305	0.102*	0.764 (3)
H31C	0.8835	0.1193	−0.0255	0.102*	0.764 (3)
O2A	0.8154 (9)	0.0724 (8)	0.0839 (2)	0.0467 (13)	0.236 (3)
C30A	0.8201 (17)	0.0081 (12)	0.0299 (7)	0.0487 (17)	0.236 (3)
H30C	0.7493	−0.0230	0.0193	0.058*	0.236 (3)
H30D	0.8721	−0.0426	0.0462	0.058*	0.236 (3)
C31A	0.848 (7)	0.042 (4)	−0.032 (2)	0.068 (3)	0.236 (3)
H31D	0.8431	−0.0104	−0.0630	0.102*	0.236 (3)
H31E	0.9207	0.0677	−0.0241	0.102*	0.236 (3)
H31F	0.7979	0.0929	−0.0497	0.102*	0.236 (3)
B1	0.4383 (3)	0.1100 (3)	0.26828 (19)	0.0346 (8)	
H1	0.3624	0.1224	0.2729	0.042*	

### Atomic displacement parameters (Å^2^)

**Table:**

	*U*^11^	*U*^22^	*U*^33^	*U*^12^	*U*^13^	*U*^23^
Ru1	0.01928 (13)	0.02232 (13)	0.03081 (14)	0.00061 (9)	0.00673 (9)	0.00169 (9)
S1	0.0393 (5)	0.0347 (4)	0.0454 (5)	−0.0073 (4)	0.0159 (4)	0.0054 (4)
S2	0.0374 (5)	0.0309 (4)	0.0439 (4)	0.0032 (3)	0.0155 (4)	−0.0018 (3)
P1	0.0202 (4)	0.0268 (4)	0.0319 (4)	0.0005 (3)	0.0060 (3)	0.0010 (3)
P2	0.0284 (4)	0.0498 (5)	0.0346 (4)	−0.0018 (4)	0.0116 (3)	−0.0028 (4)
N1	0.0237 (13)	0.0267 (13)	0.0369 (13)	0.0020 (10)	0.0065 (10)	0.0017 (11)
N2	0.0264 (13)	0.0298 (13)	0.0422 (14)	0.0062 (11)	0.0093 (11)	0.0007 (11)
N3	0.0212 (12)	0.0268 (13)	0.0352 (13)	0.0004 (10)	0.0087 (10)	−0.0018 (10)
N4	0.0188 (12)	0.0309 (13)	0.0418 (14)	−0.0007 (10)	0.0091 (10)	0.0009 (11)
N5	0.0249 (13)	0.0314 (14)	0.0374 (14)	0.0034 (11)	0.0036 (11)	0.0022 (11)
N6	0.0230 (13)	0.0311 (14)	0.0460 (15)	0.0043 (11)	0.0007 (11)	0.0036 (12)
C1	0.0347 (18)	0.0277 (16)	0.0428 (18)	−0.0022 (14)	0.0059 (14)	−0.0026 (14)
C2	0.050 (2)	0.0291 (17)	0.057 (2)	0.0076 (16)	0.0108 (17)	−0.0077 (16)
C3	0.0376 (19)	0.0357 (18)	0.050 (2)	0.0108 (15)	0.0133 (15)	−0.0032 (15)
C4	0.0298 (16)	0.0252 (15)	0.0382 (16)	−0.0014 (13)	0.0084 (13)	0.0010 (13)
C5	0.0370 (19)	0.0324 (17)	0.052 (2)	−0.0065 (15)	0.0159 (15)	0.0062 (15)
C6	0.0289 (17)	0.0407 (19)	0.0445 (18)	−0.0050 (14)	0.0137 (14)	0.0022 (15)
C7	0.042 (2)	0.0434 (19)	0.0359 (17)	0.0025 (16)	0.0016 (15)	0.0014 (15)
C8	0.046 (2)	0.054 (2)	0.043 (2)	−0.0011 (18)	−0.0077 (17)	0.0042 (17)
C9	0.0290 (18)	0.044 (2)	0.055 (2)	0.0008 (15)	−0.0078 (16)	0.0037 (17)
C10	0.0292 (16)	0.0264 (15)	0.0337 (16)	0.0032 (13)	0.0071 (12)	0.0046 (12)
C11	0.0312 (17)	0.0372 (18)	0.0366 (16)	−0.0011 (14)	0.0044 (13)	−0.0006 (14)
C12	0.046 (2)	0.0407 (19)	0.0390 (18)	−0.0037 (16)	0.0087 (15)	−0.0093 (15)
C13	0.051 (2)	0.045 (2)	0.0383 (18)	0.0001 (17)	0.0157 (16)	−0.0076 (15)
C14	0.0345 (19)	0.052 (2)	0.0451 (19)	0.0007 (16)	0.0173 (15)	−0.0001 (16)
C15	0.0321 (17)	0.0408 (18)	0.0333 (16)	−0.0035 (14)	0.0059 (13)	−0.0023 (14)
C16	0.0194 (14)	0.0302 (16)	0.0390 (16)	−0.0006 (12)	0.0008 (12)	0.0025 (13)
C17	0.0341 (18)	0.0380 (18)	0.0455 (19)	0.0100 (15)	0.0091 (15)	0.0048 (15)
C18	0.0373 (19)	0.0357 (18)	0.059 (2)	0.0068 (15)	0.0070 (16)	−0.0009 (16)
C19	0.0356 (19)	0.0291 (17)	0.066 (2)	0.0029 (15)	−0.0017 (17)	0.0054 (16)
C20	0.054 (2)	0.0369 (19)	0.047 (2)	−0.0029 (17)	0.0046 (17)	0.0127 (16)
C21	0.0406 (19)	0.0347 (18)	0.0403 (18)	−0.0024 (15)	0.0053 (15)	0.0012 (14)
C22	0.0236 (15)	0.0388 (17)	0.0331 (16)	−0.0042 (13)	0.0082 (12)	−0.0025 (13)
C23	0.0268 (17)	0.0458 (19)	0.0428 (18)	0.0000 (15)	0.0045 (14)	0.0017 (15)
C24	0.0233 (17)	0.067 (3)	0.051 (2)	−0.0018 (16)	0.0071 (15)	−0.0041 (18)
C25	0.0287 (19)	0.077 (3)	0.051 (2)	−0.0171 (19)	0.0119 (16)	−0.010 (2)
C26	0.046 (2)	0.052 (2)	0.052 (2)	−0.0224 (18)	0.0136 (17)	−0.0027 (17)
C27	0.0346 (18)	0.0390 (19)	0.0474 (19)	−0.0065 (15)	0.0049 (15)	−0.0012 (15)
O1	0.0291 (16)	0.072 (2)	0.0457 (19)	0.0045 (16)	0.0094 (15)	−0.0087 (18)
C28	0.053 (3)	0.060 (4)	0.080 (4)	0.007 (3)	0.001 (3)	−0.006 (3)
C29	0.034 (3)	0.103 (7)	0.103 (6)	0.015 (4)	0.018 (3)	−0.009 (5)
O1A	0.029 (2)	0.070 (3)	0.046 (3)	0.005 (2)	0.012 (2)	−0.007 (3)
C29A	0.034 (3)	0.103 (7)	0.103 (6)	0.015 (4)	0.018 (3)	−0.009 (5)
C28A	0.053 (4)	0.059 (4)	0.079 (5)	0.008 (4)	−0.001 (4)	−0.008 (4)
O2	0.0285 (19)	0.077 (2)	0.0354 (15)	−0.0102 (19)	0.0145 (15)	−0.0088 (15)
C30	0.048 (3)	0.053 (4)	0.040 (2)	0.001 (3)	0.003 (2)	0.002 (3)
C31	0.090 (5)	0.079 (7)	0.038 (3)	0.026 (6)	0.021 (3)	−0.010 (4)
O2A	0.032 (3)	0.076 (3)	0.036 (2)	−0.012 (3)	0.017 (2)	−0.009 (2)
C30A	0.050 (4)	0.054 (4)	0.041 (3)	0.001 (3)	0.002 (3)	0.001 (3)
C31A	0.090 (5)	0.079 (7)	0.038 (3)	0.026 (6)	0.021 (3)	−0.010 (4)
B1	0.0216 (17)	0.0350 (19)	0.047 (2)	0.0031 (15)	0.0065 (15)	0.0024 (16)

### Geometric parameters (Å, °)

**Table:**

Ru1—N3	2.086 (2)	C15—H15A	0.9300
Ru1—N1	2.088 (2)	C16—C21	1.388 (4)
Ru1—N5	2.144 (3)	C16—C17	1.390 (5)
Ru1—P1	2.3171 (8)	C17—C18	1.385 (5)
Ru1—S1	2.4540 (8)	C17—H17A	0.9300
Ru1—S2	2.4635 (8)	C18—C19	1.372 (5)
S1—P2	1.9643 (13)	C18—H18A	0.9300
S2—P2	1.9899 (12)	C19—C20	1.376 (5)
P1—C16	1.846 (3)	C19—H19A	0.9300
P1—C10	1.848 (3)	C20—C21	1.389 (5)
P1—C22	1.854 (3)	C20—H20A	0.9300
P2—O2	1.587 (3)	C21—H21A	0.9300
P2—O1A	1.591 (4)	C22—C27	1.386 (5)
P2—O2A	1.607 (4)	C22—C23	1.396 (5)
P2—O1	1.609 (3)	C23—C24	1.391 (5)
N1—C1	1.334 (4)	C23—H23A	0.9300
N1—N2	1.366 (3)	C24—C25	1.366 (6)
N2—C3	1.341 (4)	C24—H24A	0.9300
N2—B1	1.535 (5)	C25—C26	1.383 (6)
N3—C4	1.329 (4)	C25—H25A	0.9300
N3—N4	1.369 (3)	C26—C27	1.389 (5)
N4—C6	1.347 (4)	C26—H26A	0.9300
N4—B1	1.540 (4)	C27—H27A	0.9300
N5—C7	1.330 (4)	O1—C28	1.409 (5)
N5—N6	1.365 (4)	C28—C29	1.424 (6)
N6—C9	1.344 (4)	C28—H28A	0.9700
N6—B1	1.541 (5)	C28—H28B	0.9700
C1—C2	1.384 (5)	C29—H29A	0.9600
C1—H1A	0.9300	C29—H29B	0.9600
C2—C3	1.366 (5)	C29—H29C	0.9600
C2—H2A	0.9300	O1A—C28A	1.416 (6)
C3—H3A	0.9300	C29A—C28A	1.435 (6)
C4—C5	1.383 (4)	C29A—H29D	0.9600
C4—H4A	0.9300	C29A—H29E	0.9600
C5—C6	1.368 (5)	C29A—H29F	0.9600
C5—H5A	0.9300	C28A—H28C	0.9700
C6—H6A	0.9300	C28A—H28D	0.9700
C7—C8	1.388 (5)	O2—C30	1.426 (4)
C7—H7A	0.9300	C30—C31	1.446 (5)
C8—C9	1.366 (5)	C30—H30A	0.9700
C8—H8A	0.9300	C30—H30B	0.9700
C9—H9A	0.9300	C31—H31A	0.9600
C10—C11	1.396 (4)	C31—H31B	0.9600
C10—C15	1.399 (4)	C31—H31C	0.9600
C11—C12	1.390 (5)	O2A—C30A	1.420 (6)
C11—H11A	0.9300	C30A—C31A	1.438 (6)
C12—C13	1.380 (5)	C30A—H30C	0.9700
C12—H12A	0.9300	C30A—H30D	0.9700
C13—C14	1.381 (5)	C31A—H31D	0.9600
C13—H13A	0.9300	C31A—H31E	0.9600
C14—C15	1.383 (5)	C31A—H31F	0.9600
C14—H14A	0.9300	B1—H1	0.9800
			
N3—Ru1—N1	90.09 (9)	C14—C15—H15A	119.7
N3—Ru1—N5	83.57 (10)	C10—C15—H15A	119.7
N1—Ru1—N5	84.88 (10)	C21—C16—C17	117.8 (3)
N3—Ru1—P1	90.07 (7)	C21—C16—P1	123.5 (2)
N1—Ru1—P1	90.63 (7)	C17—C16—P1	118.6 (2)
N5—Ru1—P1	172.20 (7)	C18—C17—C16	121.5 (3)
N3—Ru1—S1	170.14 (7)	C18—C17—H17A	119.2
N1—Ru1—S1	93.07 (7)	C16—C17—H17A	119.2
N5—Ru1—S1	87.41 (7)	C19—C18—C17	119.9 (3)
P1—Ru1—S1	99.23 (3)	C19—C18—H18A	120.0
N3—Ru1—S2	94.61 (7)	C17—C18—H18A	120.0
N1—Ru1—S2	169.78 (7)	C18—C19—C20	119.6 (3)
N5—Ru1—S2	86.63 (7)	C18—C19—H19A	120.2
P1—Ru1—S2	98.42 (3)	C20—C19—H19A	120.2
S1—Ru1—S2	80.86 (3)	C19—C20—C21	120.6 (3)
P2—S1—Ru1	84.74 (4)	C19—C20—H20A	119.7
P2—S2—Ru1	83.95 (4)	C21—C20—H20A	119.7
C16—P1—C10	101.43 (14)	C16—C21—C20	120.6 (3)
C16—P1—C22	99.51 (14)	C16—C21—H21A	119.7
C10—P1—C22	101.07 (14)	C20—C21—H21A	119.7
C16—P1—Ru1	117.05 (10)	C27—C22—C23	118.1 (3)
C10—P1—Ru1	112.69 (10)	C27—C22—P1	119.6 (2)
C22—P1—Ru1	121.98 (10)	C23—C22—P1	122.3 (2)
O1A—P2—O2A	92.4 (6)	C24—C23—C22	120.6 (3)
O2—P2—O1	97.94 (17)	C24—C23—H23A	119.7
O2A—P2—O1	125.9 (4)	C22—C23—H23A	119.7
O2—P2—S1	118.31 (15)	C25—C24—C23	120.4 (4)
O1A—P2—S1	123.7 (4)	C25—C24—H24A	119.8
O2A—P2—S1	98.4 (4)	C23—C24—H24A	119.8
O1—P2—S1	105.05 (13)	C24—C25—C26	119.9 (3)
O2—P2—S2	115.21 (13)	C24—C25—H25A	120.0
O1A—P2—S2	122.1 (4)	C26—C25—H25A	120.0
O2A—P2—S2	105.9 (4)	C25—C26—C27	119.9 (4)
O1—P2—S2	112.03 (14)	C25—C26—H26A	120.0
S1—P2—S2	107.51 (5)	C27—C26—H26A	120.0
C1—N1—N2	106.2 (2)	C22—C27—C26	121.0 (3)
C1—N1—Ru1	134.7 (2)	C22—C27—H27A	119.5
N2—N1—Ru1	119.14 (19)	C26—C27—H27A	119.5
C3—N2—N1	109.4 (3)	C28—O1—P2	122.3 (3)
C3—N2—B1	130.7 (3)	O1—C28—C29	115.8 (6)
N1—N2—B1	119.5 (2)	O1—C28—H28A	108.3
C4—N3—N4	106.5 (2)	C29—C28—H28A	108.3
C4—N3—Ru1	135.1 (2)	O1—C28—H28B	108.3
N4—N3—Ru1	118.39 (18)	C29—C28—H28B	108.3
C6—N4—N3	109.0 (2)	H28A—C28—H28B	107.4
C6—N4—B1	130.5 (3)	C28—C29—H29A	109.5
N3—N4—B1	120.1 (2)	C28—C29—H29B	109.5
C7—N5—N6	106.2 (3)	H29A—C29—H29B	109.5
C7—N5—Ru1	135.9 (2)	C28—C29—H29C	109.5
N6—N5—Ru1	117.86 (19)	H29A—C29—H29C	109.5
C9—N6—N5	109.5 (3)	H29B—C29—H29C	109.5
C9—N6—B1	130.8 (3)	C28A—O1A—P2	119.2 (11)
N5—N6—B1	119.6 (2)	C28A—C29A—H29D	109.5
N1—C1—C2	110.6 (3)	C28A—C29A—H29E	109.5
N1—C1—H1A	124.7	H29D—C29A—H29E	109.5
C2—C1—H1A	124.7	C28A—C29A—H29F	109.5
C3—C2—C1	105.1 (3)	H29D—C29A—H29F	109.5
C3—C2—H2A	127.5	H29E—C29A—H29F	109.5
C1—C2—H2A	127.5	O1A—C28A—C29A	130 (2)
N2—C3—C2	108.8 (3)	O1A—C28A—H28C	104.7
N2—C3—H3A	125.6	C29A—C28A—H28C	104.7
C2—C3—H3A	125.6	O1A—C28A—H28D	104.7
N3—C4—C5	110.7 (3)	C29A—C28A—H28D	104.7
N3—C4—H4A	124.6	H28C—C28A—H28D	105.7
C5—C4—H4A	124.6	C30—O2—P2	120.1 (3)
C6—C5—C4	105.1 (3)	O2—C30—C31	109.8 (10)
C6—C5—H5A	127.5	O2—C30—H30A	109.7
C4—C5—H5A	127.5	C31—C30—H30A	109.7
N4—C6—C5	108.7 (3)	O2—C30—H30B	109.7
N4—C6—H6A	125.6	C31—C30—H30B	109.7
C5—C6—H6A	125.6	H30A—C30—H30B	108.2
N5—C7—C8	110.6 (3)	C30—C31—H31A	109.5
N5—C7—H7A	124.7	C30—C31—H31B	109.5
C8—C7—H7A	124.7	H31A—C31—H31B	109.5
C9—C8—C7	105.1 (3)	C30—C31—H31C	109.5
C9—C8—H8A	127.4	H31A—C31—H31C	109.5
C7—C8—H8A	127.4	H31B—C31—H31C	109.5
N6—C9—C8	108.5 (3)	C30A—O2A—P2	128.8 (11)
N6—C9—H9A	125.7	O2A—C30A—C31A	121 (3)
C8—C9—H9A	125.7	O2A—C30A—H30C	107.0
C11—C10—C15	118.3 (3)	C31A—C30A—H30C	107.0
C11—C10—P1	122.4 (2)	O2A—C30A—H30D	107.0
C15—C10—P1	119.3 (2)	C31A—C30A—H30D	107.0
C12—C11—C10	120.5 (3)	H30C—C30A—H30D	106.7
C12—C11—H11A	119.8	C30A—C31A—H31D	109.5
C10—C11—H11A	119.8	C30A—C31A—H31E	109.5
C13—C12—C11	120.4 (3)	H31D—C31A—H31E	109.5
C13—C12—H12A	119.8	C30A—C31A—H31F	109.5
C11—C12—H12A	119.8	H31D—C31A—H31F	109.5
C12—C13—C14	119.6 (3)	H31E—C31A—H31F	109.5
C12—C13—H13A	120.2	N2—B1—N4	109.7 (3)
C14—C13—H13A	120.2	N2—B1—N6	107.9 (3)
C13—C14—C15	120.5 (3)	N4—B1—N6	107.6 (3)
C13—C14—H14A	119.8	N2—B1—H1	110.5
C15—C14—H14A	119.8	N4—B1—H1	110.5
C14—C15—C10	120.7 (3)	N6—B1—H1	110.5
